# PTEN enhances the radiosensitivity of melanoma by inhibiting DNA-PKcs

**DOI:** 10.3389/fcell.2025.1712429

**Published:** 2026-01-15

**Authors:** Shengqian Zhu, Haitao Xu, Fu Shen, Fangying Chen, Yangjian Wang

**Affiliations:** 1 Department of Plastic and Reconstructive Surgery, The First Affiliated Hospital of Ningbo University, Ningbo, China; 2 Department of Radiology, Changhai Hospital, Second Military Medical University, Shanghai, China

**Keywords:** DNA damage repair, immunotherapy, irradiation, melanoma, PTEN

## Abstract

**Background:**

The phosphatase and tensin homolog (*PTEN*) is a classical tumor-suppressor gene. Its expression deficiency concurrently drives disease progression in approximately 30% of melanomas and is closely associated with radiotherapy tolerance. However, there is a lack of systematic evidence regarding whether and how PTEN regulates the radiosensitivity of melanoma.

**Methods:**

The expression of PTEN was validated using TCGA database, clinical tissue microarrays, and multiple melanoma cell lines. PTEN knockdown (PTEN-KD) and PTEN overexpression (PTEN-OE) stable cell lines were constructed using lentiviral vectors. CCK-8, colony formation assay, annexin V/PI flow cytometry, neutral comet assay, cell-cycle analysis, and Western blotting were used to assess the biological changes in cells after 0 Gy–8 Gy γ-ray irradiation (IR). A cell-derived xenograft model was established, and the tumor volume was observed after local 10 Gy IR for 28 days; in addition, H&E, Ki67, and TUNEL evaluations were performed.

**Results:**

The expression of PTEN in melanoma tissues and cell lines was significantly lower than that in normal controls. IR could induce a transient upregulation of PTEN followed by rapid downregulation. PTEN-OE significantly inhibited proliferation, reduced the clone survival rate, increased apoptosis, and weakened radiation-induced G_2_/M phase arrest; however, the opposite was true for PTEN-KD. Mechanistically, PTEN-OE inhibited the DNA-PKcs axis, reduced NHEJ-mediated rapid repair, and increased the persistent expression of γ-H2AX. PTEN-KD activated the p-ATM/p-Chk2 signaling. Animal experiments confirmed that the tumor volume in the PTEN-OE + IR group was significantly lower than that in the NC + IR group, with an expanded necrotic area, a decreased Ki67 index, and an increased TUNEL-positive rate.

**Conclusion:**

PTEN enhances the radiosensitivity of melanoma by inhibiting the DNA-PKcs signal, weakening NHEJ repair, and delaying cell-cycle recovery. PTEN can serve as a biomarker for radiotherapy response prediction and a target for sensitization intervention, providing an experimental basis for precise radiotherapy strategies for melanoma.

## Introduction

The discovery of phosphatase and tensin homolog (*PTEN*) gene has opened new avenues in tumor biology research since 1997 (Chen et al., 2018). PTEN catalyzes the dephosphorylation of phosphatidylinositol-3,4,5-trisphosphate (PIP3) by blocking the PI3K/AKT/mTOR pathway, thereby inhibiting tumor cell proliferation, inducing apoptosis, and limiting migration and invasion (Hopkins et al., 2014). However, in more than 40% of human malignant tumors, the *PTEN* gene undergoes mutations, deletions, or epigenetic silencing, resulting in the loss of its tumor suppressor function and allowing cancer cells to acquire key capabilities that drive cancer progression such as sustained proliferation and distant metastasis (Álvarez-Garcia et al., 2019). In recent years, there have been continuous efforts to elucidate the inactivation mechanisms of PTEN in various cancers, its non-classical functions, and its clinical intervention strategies, thus making this molecule one of the most important tumor-suppressor genes after p53 (Wu et al., 2024).

The inactivation of PTEN is particularly crucial in the most aggressive skin malignancy—melanoma. Approximately 30% of melanoma patients have lost or weakened PTEN function, and this event often occurs in conjunction with the *BRAF* ^V600E^ mutation, accelerating the disease to the advanced and metastatic stages (Sun et al., 2020). Research indicates that PTEN inhibits the AKT/mTOR/FRA1 signaling axis through its lipid phosphatase activity, thereby blocking the proliferation and invasion of melanoma cells; once PTEN is inactivated, this regulatory axis is relieved of inhibition, driving the malignant progression of the tumor (Xu et al., 2024). However, clinical attempts to use AKT single-target inhibitors to treat PTEN-deficient melanoma have yielded limited results, suggesting the need for further exploration of the multi-dimensional mechanism of PTEN in melanoma. Recent evidence has shown that PTEN is actively secreted by tumor cells into the microenvironment, where it reprograms tumor-associated macrophages (TAMs) to enhance the antitumor immune response of CD8^+^ T cells and NK cells, thereby presenting a novel strategy for immune microenvironment intervention in PTEN-deficient melanoma (Zhang et al., 2024).

Radiotherapy is a core and highly effective treatment method for various solid malignant tumors. However, local recurrence and radiotherapy resistance still limit its therapeutic effect. Recent evidence indicates that the deletion or inactivation of the tumor-suppressor gene *PTEN* can significantly affect the radiotherapy sensitivity of cells through multiple mechanisms. Its effect demonstrates a “double-edged sword” characteristic in different histological types and genetic backgrounds. In malignant tumors, the expression level of PTEN can be used as a biomarker for radiotherapy response prediction. Patients with PTEN deficiency may benefit from radiotherapy combined with inhibitors of the PI3K/AKT/mTOR or DNA repair pathway (Zhang et al., 2017). For individuals carrying germline PTEN mutations, vigilance against excessive toxicity from radiotherapy is necessary. Appropriate dose reduction or the use of precise radiotherapy techniques such as proton or heavy ion therapy should be considered (Tatebe et al., 2019). In view of this, a detailed elucidation of the protein characteristics of PTEN and its molecular interaction network in malignant tumors, especially melanoma, will lay a solid foundation for the development of precise treatment strategies.

## Methods

### Cell lines

Human melanoma cell lines SK-MEL-28, SK-MEL-5, and A375 and human keratinocyte cell lines HaCaT and NHEK cells were obtained from American Type Culture Collection (ATCC). SK-MEL-28, SK-MEL-5, and A375 cells were maintained in RPMI 1640 medium (Gibco) supplemented with 10% fetal bovine serum (FBS, Gibco) and 1% penicillin–streptomycin (PS). HaCaT and NHEK cells were cultured in DMEM (Gibco) supplemented with 10% FBS and 1% PS. All cells were maintained in a humidified incubator at 5% CO_2_ and 37 °C.

### Construction of PTEN-KD/OE stable cell lines

Logarithmically growing cells were used in this procedure. When cells reached 60%–70% confluency, PTEN-KD viruses were used to infect A375 cells and PTEN-OE viruses were used to infect SK-MEL-28 cells using polybrene (8 μg/mL) for 48–72 h. The cells were selected with puromycin (800 ng/mL) for 96 h. Western blot (WB) and qPCR analyses were used to confirm the successful construction of stable strains. The sequences of shRNA used against PTEN were CGT​GCA​GAT​AAT​GAC​AAG​GAA and CCA​CAA​ATG​AAG​GGA​TAT​AAA.

### Ionizing radiation (IR)

The PTEN-KD/OE cells were irradiated with ^60^Co γ-rays of 2, 4, 6, or 8 Gy at a dose rate of 1 Gy/min. Mice were subjected to localized tumor irradiation (IR) with 15 Gy at a rate of 1 Gy/min.

### Cell viability assay

The PTEN-KD/OE cells were seeded in 96-well plates at a density of 3 × 10^3^ cells per well. After culturing for 24 h, the cells were divided into groups and exposed to 0, 2, 4, 6, and 8 Gy ^60^Co γ-rays, and each group consisted of five replicates. The cells were further cultured for 24 h. A measure of 10 μL of CCK-8 reagent was added to each well, and the plates were incubated at 37 °C for 2 h. The absorbance was measured at 450 nm. The cell viability was expressed as [(experimental group OD450/control group OD450) × 100%].

### Clonogenic assay

The PTEN-KD/OE cells were inoculated into 6-well plates at a density of 200–1,000 cells/well. After incubation for 24 h, the cells were then exposed to 0, 2, 4, 6, and 8 Gy ^60^Co γ-rays, and each group consisted of five replicates. The cells were cultured for another 10–14 days and then washed with PBS, fixed with 4% paraformaldehyde for 30 min, and stained with 0.1% crystal violet for 20 min. The colonies with ≥50 cells were reported.

### Apoptosis assay

The Annexin V-APC/PI Apoptosis Detection Kit (Yeasen, China) was used to analyze cell apoptosis according to the manufacturer’s instructions. After 24 h of ^60^Co γ-ray IR at 8 Gy to the PTEN-KD/OE cells, the supernatant was collected, and the cells were digested with trypsin; each group consisted of five replicates. The cells were washed twice with PBS and then stained with annexin V-FITC/PI for 15 min in the dark. Cells were detected using a flow cytometer for calculating the apoptosis rate.

### Neutral comet assay

After exposure to 8 Gy ^60^Co γ-ray IR, the cells were immediately cultured at 37 °C and 5% CO_2_ for another 4 h. The cells were collected, washed twice with PBS, and adjusted to a density of 1 × 10^6^ cells/mL. A measure of 50 μL of the cell suspension was mixed evenly with an equal volume of 0.5% low-melting-point agarose at 37 °C. Subsequently, the mixture was added to a glass slide pre-coated with 1% normal melting-point agarose. After solidification at 4 °C for 10 min, the slide was placed in pre-cooled neutral lysis buffer (2.5 M NaCl, 1% Triton X-100, and 10 mM Tris-HCl, pH 7.5, containing 10% DMSO) and lysed at 4 °C in the dark for 2 h. After lysis, the slide was equilibrated in pre-cooled neutral electrophoresis buffer (90 mM Tris-HCl, 90 mM boric acid, and 2 mM EDTA, pH 7.5) for 20 min and then electrophoresed at 20 V and 200 mA for 25 min (4 °C). After electrophoresis, the slide was gently washed with double-distilled water and stained with SYBR Green I (1:10,000) for 20 min in the dark, and images of ≥50 cells were randomly captured under a fluorescence microscope. Each group consisted of five replicates.

### Cell-cycle analysis

PI staining was used for evaluating cell-cycle progression. After exposure to 8 Gy ^60^Co γ-rays, the cells were further cultured at 37 °C and 5% CO_2_ for 24 h and then collected. A total of 1 × 10^6^ cells were resuspended in PBS in a 15-mL centrifuge tube. A measure of 2 mL of cold anhydrous ethanol was added gradually to achieve a final concentration of 75%, and the mixture was stored at 4 °C overnight. The cells were washed twice with PBS and stained with 300 μL of PI/RNase staining solution at room temperature in the dark for 15 min. A 400-mesh sieve was used to filter the single-cell suspension, and detection was carried out using a flow cytometer. All experiments were repeated three times.

### Western blotting (WB)

After being irradiated with 8 Gy, the cells were further cultured for 24 h. The cells were lysed using RIPA lysis buffer (containing 1 mM PMSF and protease inhibitors) on ice for 30 min and then centrifuged at 12,000 × g for 15 min at 4 °C to obtain the supernatant. After quantification by the BCA method, 30 μg of protein in each well was separated using 10% SDS-PAGE and then transferred to a PVDF membrane. The membrane was incubated at room temperature with 5% skimmed milk for 1 h, washed thrice with TBST, and incubated with primary antibodies against PTEN, DNA-PKcs, p-ATM, p-ATR, p-Chk1, p-Chk2, γ-H2AX, and GAPDH (all diluted 1:1,000; Cell Signaling Technology, Danvers, MA, United States) overnight at 4 °C. After incubation, the membrane was washed thrice with TBST, incubated with HRP-labeled secondary antibody at room temperature for 1 h, and then treated with ECL for imaging.

### Immunohistochemical staining

After drying, dewaxing, hydration, and antigen retrieval, the paraffin-embedded tissue sections were processed for immunohistochemical staining. Goat serum was used to block non-specific binding sites. A dilution of the PTEN primary antibody (Sigma, SAB5701196) was added and incubated overnight at 4 °C in a humid box; PBS was used as a negative control instead of the primary antibody. After washing with PBS, the corresponding secondary antibody conjugated with HRP was added and incubated at room temperature for 30 min. After thorough washing with PBS, DAB staining solution was added for staining, and the staining time was controlled under a microscope to terminate the reaction. Finally, the cells’ nuclei were stained with hematoxylin, dehydrated, cleared, and sealed with neutral gum. All sections were observed and evaluated by two pathologists who were unaware of the grouping information under an optical microscope.

The proportion of positive cells was quantified using ImageJ. In brief, three random fields per section from three independent sections were analyzed. The positive cell count and the total cell count were recorded, and the positivity rate was determined accordingly.

### Xenograft model construction and local radiotherapy

For cell-derived xenograft (CDX) model construction, a total of 32 BALB/c-nude mice (male, 6 weeks old) were randomly divided into two groups: group 1 was subcutaneously injected with 5 × 10^6^ SK-MEL-28 NC cells per mouse in the logarithmic growth phase, and group 2 was injected with 5 × 10^6^ SK-MEL-28 OE cells per mouse. A sample size of n = 8 per group was determined based on our preliminary experiments and previous studies with similar designs. Mice were to be excluded from the final analysis if they met any of the following pre-established criteria: 1) tumor ulceration or severe infection occurred before the initiation of radiotherapy, 2) the body weight loss exceeded 20% of the initial weight during the experiment, or 3) the mouse died from causes unrelated to the tumor burden or experimental procedures (e.g., gavage error). No mice met these exclusion criteria in this study.

Tumor-bearing mice were randomly divided into four groups: NC, PTEN-OE, NC + IR, and PTEN-OE + IR. Mice in IR groups received a pelvic cavity local ^60^Co 10 Gy exposure, with the residual region of the body shielded with lead. The tumor volume was measured every 3 days by two researchers blinded to the group allocation of the mice, and then the mice were euthanized 28 days after IR or if the tumor volume reached 1,500 mm^3^. The tumor xenografts were excised, weighed, and photographed. All subsequent histological evaluations, including H&E staining, Ki67, and TUNEL analyses, were performed by pathologists who were unaware of the treatment groups.

No data points were missing from the final dataset for the primary endpoint (tumor volume and weight). All animals completed the study as per the protocol, and all collected samples were analyzed. Therefore, no imputation methods for missing data were required.

### Statistical analysis

All data analyses were conducted using Prism 10. Comparisons across multiple groups were performed using one-way ANOVA, while independent-samples Student’s t-test was used for two-group comparisons. Statistical significance was defined as a p-value<0.05. Each experiment was repeated at least three times to ensure the reproducibility of the results.

## Results

### PTEN expression is decreased in melanoma and exhibits ionizing radiation sensitization-related characteristics

**FIGURE 1:**
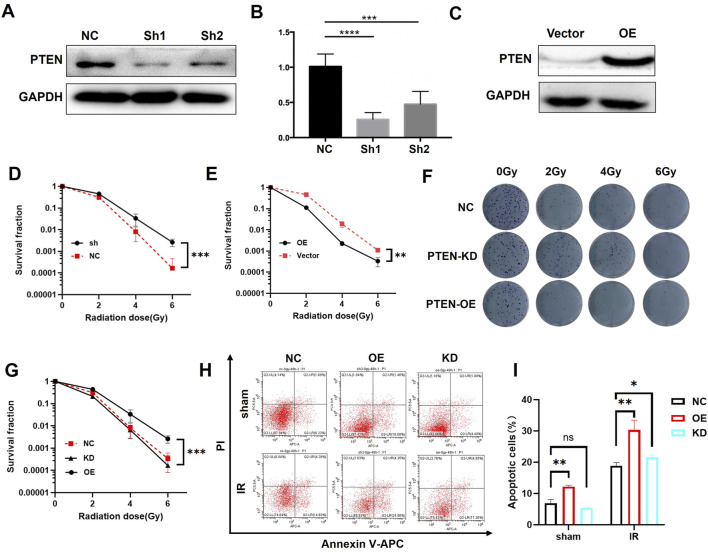
Expression of PTEN in different tissues and cells. **(A)** Expression of PTEN in melanoma tissue and normal tissue analyzed using TCGA database. **(B,C)** PTEN expression in melanoma tissues was markedly decreased compared to that in adjacent non-cancerous tissues (^****^p < 0.0001). **(D)** TLR4 expression in melanoma cells (SK-MEL-28, SK-MEL-5, and A375) was relatively lower than that in normal skin cell lines (NHEK and HaCaT). **(E)** Quantitative analysis of Figure 1D. **(F)** Expression of PTEN in A375 and SK-MEL-28 cells at different time points after IR.

As shown in Figure 1A, based on the analysis results from TCGA database, the expression of PTEN protein was downregulated in melanoma tissue compared to that in normal skin tissue. Immumohistochemical staining in patient samples revealed that PTEN expression in melanoma tissues was significantly lower than that in adjacent non-cancerous tissues (Figures 1B,C). In addition, we examined the expression levels of PTEN protein in three melanoma cell lines (A375, SK-MEL-5, and SK-MEL-28) and two keratinocyte cell lines (HaCaT and NHEK). The results indicated that the expression levels of PTEN in the melanoma cell lines were all lower than those in normal skin cell lines, which was consistent with the previous results (Figures 1D,E). After 8 Gy IR, the level of PTEN protein in the melanoma cell lines significantly increased at 0.5 h, began to decrease at 8 h, and reached the lowest level at 12 h, suggesting the responsiveness of PTEN to radiotherapy. This may be closely related to the sensitivity of melanoma to IR (Figure 1F).

**FIGURE 2:**
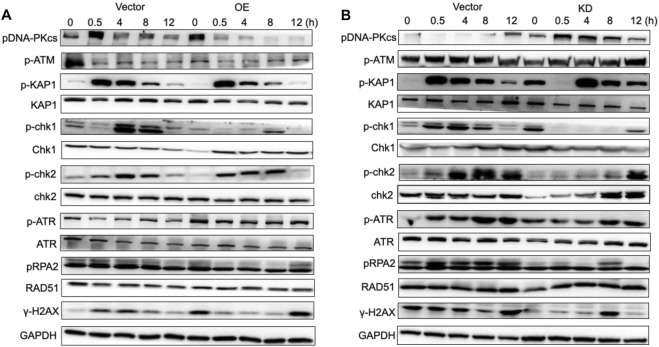
Irradiation sensitivity of melanoma cells after knockdown or overexpression of PTEN. **(A)** PTEN knockdown by shRNAs was confirmed using WB. **(B)** One-way ANOVA of Figure 2A. **(C)** PTEN overexpression was confirmed by WB. The survival rate of PTEN-KD **(D)** and PTEN-OE **(E)** were detected via CCK-8 assay. The colony formation experiment of NC, PTEN-KD, and PTEN-OE cells **(F)** and its quantitative analysis **(G)**. The flow cytometry analysis demonstrated the percentage of apoptotic cells of NC, PTEN-KD, and PTEN-OE cells **(H)** and its quantitative analysis **(I)** (^*^p < 0.05, ^**^p < 0.01, ^***^p < 0.001, and ^****^p < 0.0001).

To further investigate the role of PTEN in the radiosensitivity of melanoma, we established PTEN knockdown (PTEN-KD) and PTEN overexpression (PTEN-OE) cell lines with lentiviral transduction and verified the successful construction by WB (Figures 2A–C). Subsequently, we conducted proliferation experiments on NC, PTEN-KD, and PTEN-OE cells using the CCK-8 assay. Reducing the expression of PTEN can promote the proliferation of melanoma cells (Figure 2D), while the overexpression of PTEN reduces the proliferation rate of melanoma cells (Figure 2E). Furthermore, the colony formation experiment demonstrated that PTEN-KD cells exhibited higher colony formation ability than NC cells after receiving IR treatment, while PTEN-OE cells showed the opposite result (Figures 2F,G). The flow cytometry result in Figures 2H,I shows that after 8 Gy IR, the apoptosis rate of PTEN-OE cells was higher than that of the NC group. The above experiments have demonstrated that PTEN plays a significant role in the radiotherapy sensitivity of melanoma cells. Upregulation of PTEN expression can enhance the radiotherapy sensitivity of melanoma cells, causing a reduction in cell proliferation and an increase in apoptosis after IR.

### PTEN enhances the radiosensitivity in melanoma cells

**FIGURE 3:**
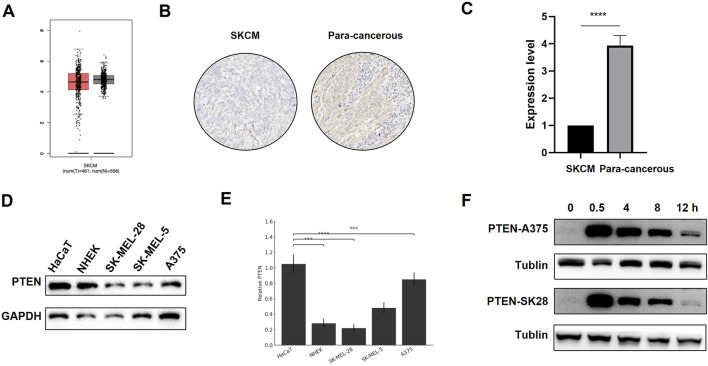
DNA repair ability of melanoma cells after PTEN overexpression. **(A)** Comet assays showed that PTEN-OE cells exhibited significantly longer tails post-irradiation. **(B,C)** Quantitative analysis of the comet assay. **(D)** Cell-cycle progression indicated that PTEN-OE mitigated the radiation-induced G2/M phase arrest. **(E)** Quantitative analysis of the cell-cycle distribution (^*^p < 0.05, ^**^p < 0.01, and ^***^p < 0.001).

As shown in Figures 3A–C, the neutral comet assay demonstrated that after 8 Gy IR, the comet tails of cells in the PTEN-OE group were significantly longer than those in the NC group, and the quantitative tail moment was significantly increased (p < 0.01), suggesting that high expression of PTEN can effectively increase DNA double-strand break damage. Cell-cycle analysis (Figures 3D,E) further revealed that at each time point after IR, the proportion of cells in the G_2_/M phase in the PTEN-OE group was consistently less than that in the NC group, which indicated the alleviated G2/M phase arrest triggered by radiation exposure. The proportions of cells in the G_2_/M phase at 6 h, 12 h, and 24 h decreased by approximately 40%, 35%, and 30%, respectively (p < 0.001), indicating that PTEN overexpression enhances the recovery of the cell cycle by weakening the activation of the G_2_/M checkpoint induced by radiation, thereby increasing the radiosensitivity of melanoma cells.

### PTEN decreased the activation of DNA-PKcs to inhibit DNA repair processes

**FIGURE 4:**
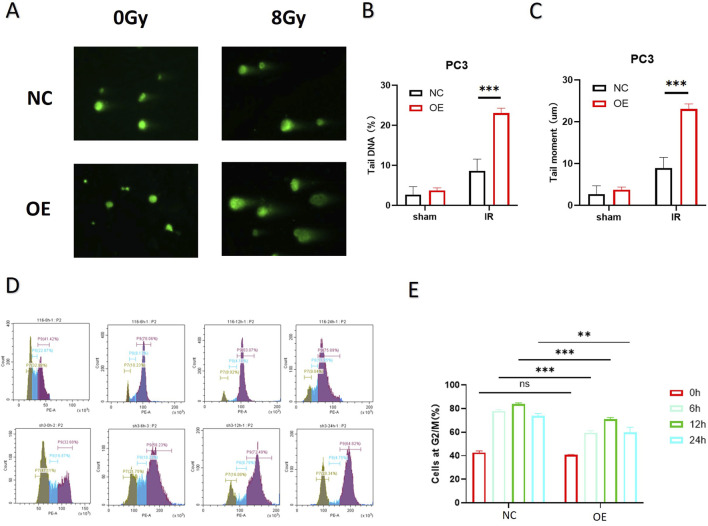
Effect of PTEN in the NHEJ pathway. **(A)** In the PTEN-OE group, DNA-PKcs activation was blocked. The activation of p-ATM and p-Chk2 was markedly suppressed. **(B)** In the PTEN-KD group, DNA-PKcs activation was expressed. The activation of p-ATM and p-Chk2 was markedly upregulated.

The homologous recombination (HR) pathway and non-homologous end joining (NHEJ) pathway are two major DNA damage repair pathways. The HR pathway uses sister chromatids as templates to precisely repair DNA double-strand breaks during the S/G_2_ phase with high fidelity but at a relatively long time-consuming process; in contrast, the NHEJ pathway directly “stitches” the broken ends through the Ku–DNA-PKcs–LIG4 complex, completing the repair quickly in all stages of the cell cycle. Although it is prone to introducing small fragment mutations, it can effectively avoid chromosomal abnormalities and cell death. To investigate the involvement of PTEN in the signaling pathway associated with the DNA damage response (DDR), proteins from NC, PTEN-OE, and PTEN-KD at time points 0, 0.5, 4, 8, and 12 h post-IR were collected following exposure to 8 Gy IR. The findings indicated that in the NC group, activation of the DNA-PKcs pathway was triggered after IR, with the level of activation progressively decreasing over time up to 12 h (Figure 4A). However, in the PTEN-OE group, DNA-PKcs activation was significantly decreased. Moreover, the activation of p-ATM and p-Chk2 was markedly suppressed in the PTEN-KD group (Figure 4B). As a marker of DNA damage, the level of γ-H2AX remained elevated at 12 h after IR in the PTEN-OE group, aligning with the results of the comet assays, as shown in Figure 3A. These observations suggest that PTEN inhibited the activation of the NHEJ signaling pathway.

### High PTEN expression promotes PD-L1 inhibitor therapy response and is associated with neutrophil capture

We included a total of 10 patients who had been diagnosed with melanoma through pathological examination. All patients received neoadjuvant chemotherapy combined with PD-L1 therapy followed by radical surgery. Melanoma specimens were divided into the pathologic complete response group (four cases) and the non-responsive group (six cases) according to the evaluation by pathologists.

**FIGURE 5:**
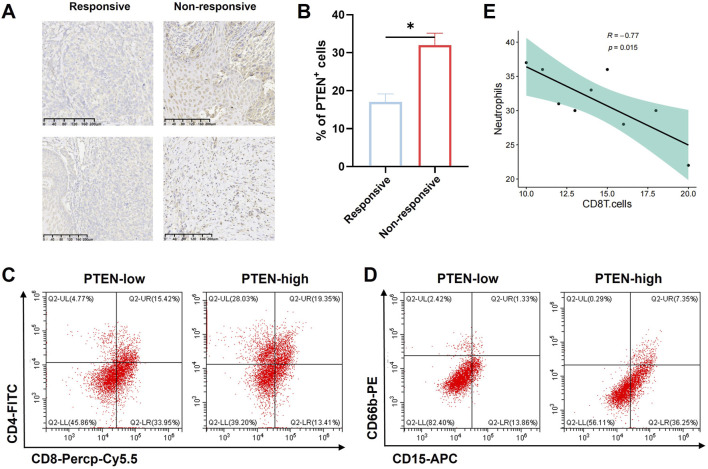
Influence of PTEN on tumor immune response. **(A,B)** PTEN showed high expression in the PD-L1 response group from melanoma samples. The number of CD4^+^ cells **(C)** and neutrophils in the innate immune cells (CD45^+^CD15^+^CD66b^+^) **(D)** was significantly higher in the PTEN-high group. **(E)** Correlation analysis showed that the number of neutrophils was significantly negatively correlated with the number of CD8^+^ T cells (ns, no significance, ^**^p < 0.01, and ^***^p < 0.001).

Immunohistochemical staining was utilized to examine PTEN expression in the above specimens. The results revealed elevated expression of PTEN in the response group (Figures 5A,B). Moreover, flow cytometric analysis of these melanoma samples demonstrated a notable increase in the count of CD4^+^ T cells (Figure 5C) and neutrophils (CD45^+^CD15^+^CD66b^+^ cells) (Figure 5D) among innate immune cells in PTEN-high patients. Furthermore, correlation analysis indicated a significant negative association between the number of neutrophils and CD8^+^ T cells (Figure 5E).

### PTEN confers radiosensitization *in vivo*

**FIGURE 6:**
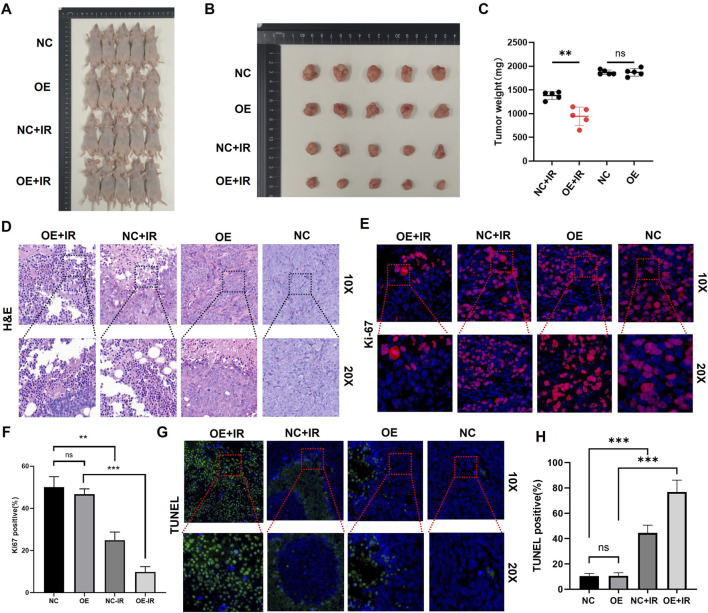
*In vivo* experiments on the effect of PTEN on the radiosensitivity of melanoma. **(A)** Representative images captured before autopsy (n = 5). **(B)** Tumor size from different groups after radiotherapy (^*^p < 0.05). **(C)** Tumor weights were measured across various groups. **(D)** H&E staining of tumors in four groups. Ki67 immunofluorescence staining **(E)** and quantitative assessment **(F)** of tumors in the four groups. TUNEL immunofluorescence staining **(G)** and quantitative assessment **(H)** of tumors in the four groups.

In the CDX model constructed using SK-MEL-28 cells, when the tumors reached approximately 100 mm^3^, they were randomly divided into four groups: NC, NC + IR, OE, and OE + IR. The IR group received a single 10 Gy local IR. According to the ethical endpoint (tumor volume > 1,500 mm^3^ or 28 days post-IR), the mice were euthanized, and the tumor tissues were collected and weighed. The results demonstrated that tumors in the OE + IR group had significantly lower weight than those in the three other groups (Figures 6A–C). H&E staining of the tumor tissues revealed that the necrotic area in the OE + IR group was significantly expanded (Figure 6D). Moreover, Ki67 staining showed a significantly decreased proliferation index in the OE + IR group (Figures 6E,F), and TUNEL staining showed a significantly increased apoptosis rate in the OE + IR group (Figures 6G,H). These results suggest that overexpression of PTEN can significantly enhance the radiotherapy sensitivity of melanoma *in vivo*, likely through its influence on apoptotic and proliferative pathways following IR. The results provide experimental evidence supporting PTEN as a potential target for radiosensitization in melanoma therapy.

## Discussion

Melanoma accounts for only 4% of all skin cancers, but it contributes to 80% of deaths caused by skin cancers (Wang et al., 2021). The traditional view holds that it is “naturally resistant” to photon radiotherapy. However, with the adoption of large fractionation (≥6 Gy per session), stereotactic radiotherapy (SRS/SBRT), or proton and heavy-ion technologies in recent years, the local control rate has increased to 60%–80%. More importantly, preclinical studies have confirmed that radiotherapy can activate CD8^+^ T cells through the “distant effect”, working in synergy with PD-1 inhibitors. The emergence of this combined “radiotherapy + immunotherapy/targeted therapy” approach shows promise for significantly improving the survival of advanced-stage patients (Khan et al., 2011). Additionally, physical–chemical sensitization strategies such as cold plasma and ICG photosensitizers have been proven to reduce the radioresistance of melanoma cells (Momeni et al., 2023). However, identifying the truly beneficial population remains a bottleneck. Molecular marker-guided precise radiotherapy has become a new direction.

PTEN, as a lipid phosphatase, inhibits proliferation and promotes apoptosis through the PI3K/AKT/mTOR axis and maintains genomic stability. In solid tumors, the inactivation of PTEN occurs through mutations, deletions, methylation, and miRNA inhibition. The incidence rate is as high as 80% in endometrial cancer and 5%–20% in melanoma (Aguissa-Touré and Li, 2012). In the nasopharyngeal carcinoma model, low expression or knockdown of PTEN can activate the PI3K/AKT/β-catenin axis, enhance the characteristics of tumor stem cells (CSCs), and thereby increase the resistance of cells to 4 Gy IR; PI3K inhibitor LY294002 or AKT inhibitor perifosine can reverse this resistance, suggesting that the PTEN/PI3K/AKT pathway is a key node regulating radiotherapy sensitivity (Zhang et al., 2017). Contrary to the “acquired resistance” of tumor cells, the loss of PTEN function is manifested as high radiosensitivity in normal tissues. Patients with Cowden syndrome (CS) carry germline PTEN mutations, and their skin fibroblasts are more sensitive to ionizing radiation in *in vitro* experiments; clinically, these patients experience unexpected normal tissue toxicity after radiotherapy, suggesting that the absence of PTEN leads to a decrease in the efficiency of DNA double-strand break repair, thereby increasing the risk of radiotherapy damage to normal tissues (Tatebe et al., 2019).

In melanoma, the absence of PTEN often co-occurs with *BRAF* ^V600E^, forming a “MAPK + PI3K″ dual activation pattern that drives invasion and metastasis. Animal models have confirmed that *BRAF* ^V600E^/PTEN^−/−^ mice can develop invasive melanoma within 3–4 weeks (Ya et al., 2015). Clinical pathological studies have also shown that patients with low PTEN expression are more likely to develop distant metastasis and resistance to BRAF inhibitors; conversely, those with high PTEN expression have abundant immune infiltration, suggesting that PTEN can serve as an independent factor for predicting the efficacy of targeted therapy and immunotherapy (Dankort et al., 2009).

In addition to its classical tumor-suppressor function, recent studies have revealed that PTEN can regulate the transcription of RAD51 through nuclear translocation, affecting homologous recombination repair, suggesting its dual role in the DNA damage response (Ming and He, 2012). The determinants of radiotherapy sensitivity include DNA repair capacity, cell cycle distribution, and microenvironment. HR and NHEJ pathways are the two main pathways involved in DNA double-strand break repair. Inhibiting PI3K/AKT can downregulate the activity of DNA-PKcs and reduce the efficiency of NHEJ, thereby sensitizing the cells (Sancar et al., 2004). The comet assay in cells conducted by our research group revealed that overexpression of PTEN led to an increase in the tail moment after 8 Gy IR, suggesting an increase in DSBs. The CDX model in nude mice further confirmed that overexpression of PTEN combined with 10 Gy local IR could significantly inhibit the proliferation rate of tumors and the apoptosis of tumors was significantly increased.

This study provides compelling evidence that PTEN enhances the radiosensitivity of melanoma by reducing DNA-PKcs activation and sustaining DSB signaling, consistent with impaired NHEJ. This finding is largely consistent with the known role of PTEN in regulating DNA damage response in other cancers. Moreover, the observed correlation between high PTEN expression and improved response to PD-L1 immunotherapy aligns with growing evidence that PTEN loss creates an immunosuppressive microenvironment, though the link to neutrophil infiltration and CD8^+^ T-cell dynamics provides a novel insight into potential immune-mediated mechanisms of radiosensitization.

Despite these strengths, several limitations should be noted. First, the study relies heavily on CDX models in immunodeficient mice, which preclude the evaluation of PTEN’s role in modulating antitumor immunity in the context of radiotherapy. Second, although PTEN is shown to suppress DNA-PKcs activation, the direct molecular interaction—whether through physical binding, phosphatase activity, or transcriptional regulation—remains unclear. Further co-immunoprecipitation or phosphatase-dead mutant experiments would help clarify the mechanism. Third, the clinical sample size for immune correlation analysis is relatively small, limiting the generalizability of the findings regarding neutrophils and T-cell subsets. Finally, although the study suggests that PTEN may serve as a biomarker for radiotherapy response, prospective validation in larger cohorts receiving uniform radiotherapy regimens is necessary before clinical translation.

## Conclusion

This study has systematically demonstrated that PTEN is both a tumor-suppressor gene and a key switch for radiotherapy sensitivity in melanoma. Cell experiments showed that the downregulation of PTEN expression is closely related to the progression of melanoma. Functional experiments indicated that overexpression of PTEN significantly enhanced the radiotherapy sensitivity of cells, which manifested as inhibition of proliferation, decreased clonogenic ability, increased apoptosis, and weakened G_2_/M phase arrest. Mechanistically, PTEN inhibits the DNA-PKcs axis and weakens the rapid repair of NHEJ, leading to the accumulation of DNA double-strand breaks; meanwhile, it downregulates the p-ATM/p-Chk2 signal, delaying the cell-cycle recovery. The CDX mouse model confirmed that PTEN overexpression combined with 10 Gy local IR significantly inhibited tumor growth, increased necrosis and apoptosis, and decreased the Ki67 index. In summary, PTEN can be used as a molecular marker for predicting the radiotherapy response of melanoma. Its overexpression or reactivation provides new theoretical basis and therapeutic targets for improving radiotherapy efficacy and formulating precise combined strategies.

## Data Availability

The raw data supporting the conclusions of this article will be made available by the authors, without undue reservation.

## References

[B1] Aguissa-Touré A. H. Li G. (2012). Genetic alterations of PTEN in human melanoma. Cell Mol. Life Sci. 69 (9), 1475–1491. 10.1007/s00018-011-0878-0 22076652 PMC11114653

[B2] Álvarez-Garcia V. Tawil Y. Wise H. M. Leslie N. R. (2019). Mechanisms of PTEN loss in cancer: it's all about diversity. Semin. Cancer Biol. 59, 66–79. 10.1016/j.semcancer.2019.02.001 30738865

[B3] Chen C. Y. Chen J. He L. Stiles B. L. (2018). PTEN: tumor suppressor and metabolic regulator. Front. Endocrinol. (Lausanne) 9, 338. 10.3389/fendo.2018.00338 30038596 PMC6046409

[B4] Dankort D. Curley D. P. Cartlidge R. A. Nelson B. Karnezis A. N. Damsky W. E. Jr (2009). Braf(V600E) cooperates with pten loss to induce metastatic melanoma. Nat. Genet. 41 (5), 544–552. 10.1038/ng.356 19282848 PMC2705918

[B5] Hopkins B. D. Hodakoski C. Barrows D. Mense S. M. Parsons R. E. (2014). PTEN function: the long and the short of it. Trends Biochem. Sci. 39 (4), 183–190. 10.1016/j.tibs.2014.02.006 24656806 PMC4043120

[B6] Khan M. K. Khan N. Almasan A. Macklis R. (2011). Future of radiation therapy for malignant melanoma in an era of newer, more effective biological agents. Onco Targets Ther. 4, 137–148. 10.2147/OTT.S20257 21949607 PMC3176173

[B7] Ming M. He Y. Y. (2012). PTEN in DNA damage repair. Cancer Lett. 319 (2), 125–129. 10.1016/j.canlet.2012.01.003 22266095 PMC3326178

[B8] Momeni S. Shanei A. Sazgarnia A. Azmoonfar R. Ghorbani F. (2023). Increased radiosensitivity of melanoma cells through cold plasma pretreatment mediated by ICG. J. Radiat. Res. 64 (5), 751–760. 10.1093/jrr/rrad042 37586714 PMC10516736

[B9] Sancar A. Lindsey-Boltz L. A. Unsal-Kaçmaz K. Linn S. (2004). Molecular mechanisms of mammalian DNA repair and the DNA damage checkpoints. Annu. Rev. Biochem. 73, 39–85. 10.1146/annurev.biochem.73.011303.073723 15189136

[B10] Sun H. Meng Y. Yao L. Du S. Li Y. Zhou Q. (2020). Ubiquitin-specific protease 22 controls melanoma metastasis and vulnerability to ferroptosis through targeting SIRT1/PTEN/PI3K signaling. MedComm 5 (8), e684. 10.1002/mco2.684 39135915 PMC11318338

[B11] Tatebe K. Chmura S. J. Connell P. P. (2019). Elevated radiation therapy toxicity in the setting of germline PTEN mutation. Pract. Radiat. Oncol. 9 (6), 492–495. 10.1016/j.prro.2019.06.001 31185301 PMC6832791

[B12] Wang Q. Huang C. Hu Y. Yan W. Gong L. (2021). Preliminary screening and correlation analysis for lncRNAs related to radiosensitivity in melanoma cells by inhibiting glycolysis. Zhong Nan Da Xue Xue Bao Yi Xue Ban. 46 (6), 565–574. 10.11817/j.issn.1672-7347.2021.200549 34275924 PMC10930195

[B13] Wu D. Huang C. Guan K. (2024). Mechanistic and therapeutic perspectives of miRNA-PTEN signaling axis in cancer therapy resistance. Biochem. Pharmacol. 226, 116406. 10.1016/j.bcp.2024.116406 38969299

[B14] Xu X. Bok I. Jasani N. Wang K. Chadourne M. Mecozzi N. (2024). PTEN lipid phosphatase activity suppresses melanoma formation by opposing an AKT/mTOR/FRA1 signaling axis. Cancer Res. 84 (3), 388–404. 10.1158/0008-5472.CAN-23-1730 38193852 PMC10842853

[B15] Ya Z. Hailemichael Y. Overwijk W. Restifo N. P. (2015). Mouse model for pre-clinical study of human cancer immunotherapy. Curr. Protoc. Immunol. 108, 20–43. 10.1002/0471142735.im2001s108 25640991 PMC4361407

[B16] Zhang G. Wang W. Yao C. Zhang S. Liang L. Han M. (2017). Radiation-resistant cancer stem-like cell properties are regulated by PTEN through the activity of nuclear β-catenin in nasopharyngeal carcinoma. Oncotarget 8 (43), 74661–74672. 10.18632/oncotarget.20339 29088815 PMC5650370

[B17] Zhang C. Ma H. M. Wu S. Shen J. M. Zhang N. Xu Y. L. (2024). Secreted PTEN binds PLXDC2 on macrophages to drive antitumor immunity and tumor suppression. Dev. Cell 59 (23), 3072–3088.e8. 10.1016/j.devcel.2024.08.003 39197453

